# Investigations into and Experiments upon the Physiological and Therapeutical Properties of Curare

**Published:** 1869-04-15

**Authors:** Auguste Voisin, Henry Lionville

**Affiliations:** La Gazette Médicale; La Gazette Médicale


### FOREIGN ITEMS.

### *Investigations into and Experiments upon the Physiological and Therapeutical Properties of Curare.*

BY MM. AUGUSTE VOISIN AND HENRY LIONVILLE—FROM LA GAZETTE MÉDICALE.

The authors have thus epitomized the principal results of their labors: "It appears to us interesting to indicate some of the most prominent facts which curaric influence, intoxication or poisoning of animals have permitted us to observe.

"From the first, the effect manifested in the economy by the curare has always appeared to us characteristic, and we have never, although observing closely, and varying the doses and the animals, had to confound it with the effect resulting from other poisons, as strychnia, for example, since it is to this that some authors, MM. Martin Magron and Buisson, especially, have compared it heretofore.

The little clonic convulsions which are observed, the fibrillary trembling, which, localized sometimes, are more frequently generalized, this minutely tremulous condition of the body which augments by external impressions received by the animal, and which increases, so to speak, gradually, and extends itself over the whole body, betraying itself by the fibrillary trembling of the muscles, or by the undulatory state of the hairy skin, which seems as though agitated gently by the wind; all these appearances, in a word, are not the tonic, forcible, abrupt, sudden convulsions manifested by an animal under the influence of strychnia. We have not, under the influence of curare, that sort of violent discharge which increases by the most feeble exterior excitement, a slight touch, the least noise, and which curves, in an irresistible manner, the whole body of the animal, like a cord which bends suddenly the two extremities of an arc. The aggregation of the curaric phenomena, shivering, is one of the manifestations of fever.

Another point which has seemed to us to deserve attention is the possible modifications of temperature during the curaric phenomena.

We have observed a very marked elevation of temperature in the rectum of animals in those instances in which the doses were poisonous. There was indicated, indeed, an augmentation of three to four degrees.

However, the exterior temperature of the whole body of the animal has never seemed to us to exhibit very perceptible modifications, even in connection with the manifestation of discoloration and *heat of the ears* upon which M. Claude Bernard has especially insisted.

But when we have found this latter phenomenon unmistakably manifest in our patients, it was always associated with a very decided augmentation of the *redness and heat of the face*, and this facial redness and heat have always seemed to us the predominant characteristic.

At the same time we may detect noticeable effects upon the circulation, as we believe, contrary to the opinion of certain physiologists, developed during the earlier stages, and subsequently modified considerably if the dose be more active.

In reference to the visual apparatus, we have detected those disturbances which are indicated by modifications in the diameter of the pupils, and we have seen the pupil doubled in size under the influence of curare.

But if the dose be still more active, this augmentation is also more considerable, and soon attains to the double size; then occurs this curious phenomenon: to the augmentation of the pupil succeeds, almost suddenly, a diminution, and even a constriction.

These disturbances have, under our observation, always appeared to accompany another phenomenon, which, we think, has not, hitherto, sufficiently attracted the attention of experimenters; it is the *double exophthalmia* which supervenes after the employment of active poisonous doses, and which in four of our experiments is recorded with minute detail.

Moreover, we have observed that these different disturbances, those of the pupil, of the injected sclerotic, of the exophthalmic eyes (which we have taken care to complete by examination of the modifications in the sight itself. *Diplopia, derangements of accommodation*, observable only in patients capable of describing it accurately, were concomitant phenomena, and might very reasonably be assumed to be simultaneous, and, as it were, associated expressions of the same general state (disturbance of the vaso-motor system, and paralysis of the muscles of the eye).

In reference to the nervous system, the authors have determined anew that curare is a poison of motor nerves; that muscular irritability is preserved intact, and that death from curare poisoning occurs only from abolition of motor power in the entire economy.

